# High Diffusion Permeability of Anion-Exchange Membranes for Ammonium Chloride: Experiment and Modeling

**DOI:** 10.3390/ijms23105782

**Published:** 2022-05-21

**Authors:** Ekaterina Skolotneva, Kseniia Tsygurina, Semyon Mareev, Ekaterina Melnikova, Natalia Pismenskaya, Victor Nikonenko

**Affiliations:** Membrane Institute, Kuban State University, 149 Stavropolskaya St., 350040 Krasnodar, Russia; ek.skolotneva@gmail.com (E.S.); kseniya_alx@mail.ru (K.T.); mareev-semyon@bk.ru (S.M.); ekaterinabelashova23@gmail.com (E.M.); n_pismen@mail.ru (N.P.)

**Keywords:** ion-exchange membrane, diffusion permeability, weak electrolyte, ammonium chloride, simulation

## Abstract

It is known that ammonium has a higher permeability through anion exchange and bipolar membranes compared to K^+^ cation that has the same mobility in water. However, the mechanism of this high permeability is not clear enough. In this study, we develop a mathematical model based on the Nernst–Planck and Poisson’s equations for the diffusion of ammonium chloride through an anion-exchange membrane; proton-exchange reactions between ammonium, water and ammonia are taken into account. It is assumed that ammonium, chloride and OH^−^ ions can only pass through membrane hydrophilic pores, while ammonia can also dissolve in membrane matrix fragments not containing water and diffuse through these fragments. It is found that due to the Donnan exclusion of H^+^ ions as coions, the pH in the membrane internal solution increases when approaching the membrane side facing distilled water. Consequently, there is a change in the principal nitrogen-atom carrier in the membrane: in the part close to the side facing the feed NH_4_Cl solution (pH < 8.8), it is the NH_4_^+^ cation, and in the part close to distilled water, NH_3_ molecules. The concentration of NH_4_^+^ reaches almost zero at a point close to the middle of the membrane cross-section, which approximately halves the effective thickness of the diffusion layer for the transport of this ion. When NH_3_ takes over the nitrogen transport, it only needs to pass through the other half of the membrane. Leaving the membrane, it captures an H+ ion from water, and the released OH^−^ moves towards the membrane side facing the feed solution to meet the NH_4_^+^ ions. The comparison of the simulation with experiment shows a satisfactory agreement.

## 1. Introduction

Nitrogen extraction from wastewater is crucial for achieving sustainable development goals. The presence of nitrogen in wastewater causes significant harm to the environment. Nitrogen removal can prevent the waters from eutrophication [1], leading to the degradation of water quality, increased N_2_O emissions (which is a greenhouse gas and has the highest impact on ozone depletion among other ozone-depleting gazes [2]) produced by bacteria into the atmosphere [3] and the occurrence of harmful algae blooms [4]. On the other hand, ammonia nitrogen is a key component of fertilizers [5], which are needed more and more to produce enough rations to feed the growing global population and overcome hunger [6]. Nowadays, ammonia is synthesized commercially using the Haber–Bosch process, which is a highly energy-intensive technology [7]. There are statistical data [8,9] that predict that 1–2% of world energy consumption will be spent on the Haber–Bosch process in the coming years. At the same time, animals or humans absorb only 16% of nitrogen from fertilizers, and the rest is released into the atmosphere or hydrosphere. Therefore, the ammonia recuperation from wastewater is a promising source of ammonia. In addition, ammonia salts have significant application potential for carbon-free energy storage and electrical power generation [10,11,12,13]. 

**Figure 1:**
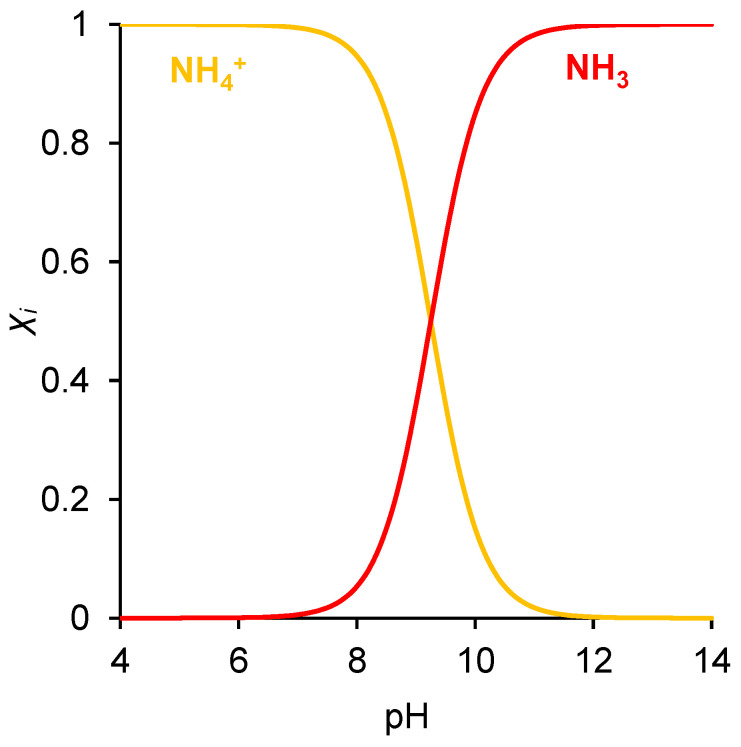
The distribution of molar fraction (X_i_) of NH_4_^+^ and NH_3_ as a function of pH.

To date, various methods of ammonia extraction have been established (chemical precipitation/crystallization, liquid-gas stripping) or intensively developed (adsorption, bio-electrochemical methods and electrodialysis (ED)) [14,15]. Among these technologies, ED stands out among others as it allows for the attainment of commercially attractive concentrates using feed solutions with low concentrations [16,17,18]. However, by its chemical nature, ammonium is an ampholyte, i.e., it participates in protonation–deprotonation reactions and can change its structure and charge depending on the pH value (Figure 1). Thus, its behavior in the electromembrane systems with ion-exchange membranes (IEMs) is more complicated and less predictable than that of strong electrolyte solutions, such as NaCl, KCl and NaNO_3_.

There are a number of studies pointing to increased water splitting at anion-exchange membranes (AEMs) in ammonia-containing solutions [19,20,21,22]; in addition, the AEM permeability for ammonium ions is higher than for other anions [22,23]. The results of these studies allow us to suggest that the specific behavior of AEMs in ammonium-containing solutions is due to protonation–deprotonation reactions involving nitrogen ammonia species, which are coupled with a pH shift in these membranes in relation to the pH of the external solution. The latter is caused by the Donnan exclusion of protons as coions from AEMs [24]. This assumption is confirmed by the fact that, in systems with bipolar membranes, inside which the pH shift is even more significant than in systems with monopolar membranes, the diffusion of ammonium through the anion-exchange layer is even more considerable [25,26,27]. 

It should also be noted that ammonia molecules are very similar in their properties to water molecules. Indeed, both molecules have the same molecular orbital hybridization, both are polar and have similar values of size (2.60 Å for ammonia and 2.65 Å for water) and dipole moments (1.47 D for ammonia and 1.85 D for water [28]), and both are able to form hydrogen bonds. This resemblance leads to the fact that ammonia can penetrate through biological membranes, which are selective to water transport [29,30,31]. Moreover, there are studies showing that, in ammonia media, the same specific mechanism of proton transfer as in water is possible. The Grotthus-type proton hops along an “ammonia wire” involving NH_3_ molecules were proved in [32,33].

Despite the experimental evidence of the unusual behavior of systems with ammonia-containing solutions, there are very few theoretical studies in this field. In the previous work conducted by our group [34], the high-ammonium transport through the AEM is explained by a mechanism similar to the facilitated diffusion, or carrier-mediated diffusion of various substances, e.g., amino acids, which is extensively described in the literature [35,36,37,38]. It was established that, due to the Donnan exclusion of H^+^ ions, the pH inside the AEM increases [24]. Therefore, when the NH_4_^+^ ions being coions for an AEM enter the membrane, a part of them loses its charge and is transformed into NH_3_ molecules, which are not excluded from the membrane. Therefore, nitrogen transfer through an AEM is not only possible with NH_4_+ ions, but NH_3_ molecules can also carry it. For the examination of this hypothesis, a one-dimensional stationary mathematical model of ammonium chloride transport through AEM was developed on the basis of the Nernst–Planck equation and the local electroneutrality assumption; protonation–deprotonation reactions inside the membrane were taken into account. The conditions of the local ion-exchange equilibrium at the solution/membrane interfaces and chemical equilibrium at any point were assumed. The latter implies that the rate constants of the protonation–deprotonation reactions are infinitely large. A qualitative agreement between the experimental data and the results of the simulation was found. 

A similar mechanism of ammonium transfer, taking into account protonation–deprotonation reactions, but through a cation-exchange membrane (CEM), was experimentally and theoretically studied by Liu et al. [39]. During the experiment, it was found that the ammonium concentration in the anode chamber decreased due to its transfer through the CEM to the cathode chamber, but the ammonium concentration in the cathode chamber remained almost constant and close to zero. The suggested explanation of this phenomena was the deprotonation of ammonium ions and their transformation in the ammonia molecules in the cathode chamber where a high value of pH was due to OH^−^ generation onthis electrode. However, the authors did not observe the back diffusion of nitrogen in the ammonia form into the anode chamber. Mathematical modeling showed that, inside the membrane, pH increases from an acidic value at the interface with the anolyte (where H^+^ ions are generated at the anode) to an alkaline value at the interface with the catholyte. Therefore, ammonia molecules, passing through the membrane and entering its acidic region, are protonated and turned into the ammonia ions, which are transferred back to the cathode chamber. Using the mathematical model, the authors also showed that, at low and medium values of electric currents, the diffusion of ammonium through the CEM prevailed over migration. The assumptions made in the model were similar to those used in Ref. [33]; in particular, the conditions of local electrical neutrality and chemical equilibrium were accepted.

The purpose of this work is to clarify the reasons for the high diffusion permeability of AEMs in an ammonium chloride solution. We present experimental data and a novel 1D stationary mathematical model of the weak electrolyte transport in a membrane system to explain the phenomenon. As in the previous theoretical works [34,39], the Nernst–Planck equations are used, taking into account the protonation–deprotonations reactions between NH_4_^+^ and NH_3_. However, instead of the local electroneutrality assumption, we use the Poisson’s equation; instead of the chemical equilibrium assumption, we apply equations describing the kinetics of chemical reactions with finite rate constants. A new assumption is applied: ammonium, chloride and hydroxyl ions can only pass through the hydrophilic pores of an AEM, while ammonia can diffuse both through the pores and through fragments of the membrane matrix that do not contain water.

## 2. Results and Discussions

### 2.1. Diffusion Permeability and Conductivity of IEMs

The integral diffusion permeability coefficient, *P*, of an ion-exchange membrane is a proportionality factor in the following equation determining the diffusion flux density, *j*, of dissolved salt through the membrane in conditions where the membrane is bathed by a (feed) solution of concentration *c* on one side and by distilled water on the other:

(1) $$\;j=P\frac{c}{d}$$

where *d* is the membrane thickness. *P* is found by measuring the value of *j* for a given value of *c* (see Section 3.2 for more details). Instead of the difference in concentrations on both sides of the membrane, the numerator contains only the concentration of the feed (bulk) solution, since there is pure water on the other membrane side and the effect of diffusion layers in the solution is assumed to be negligible. 

**Figure 2:**
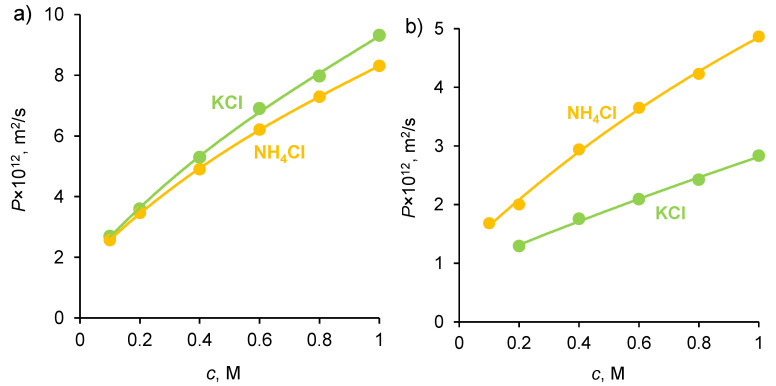
Integral diffusion permeability coefficient of CMX (**a**) and AMX (**b**) membranes in KCl and NH_4_Cl solutions. The dots are experimental data; the solid lines are presented to lead the eye.

**Figure 3:**
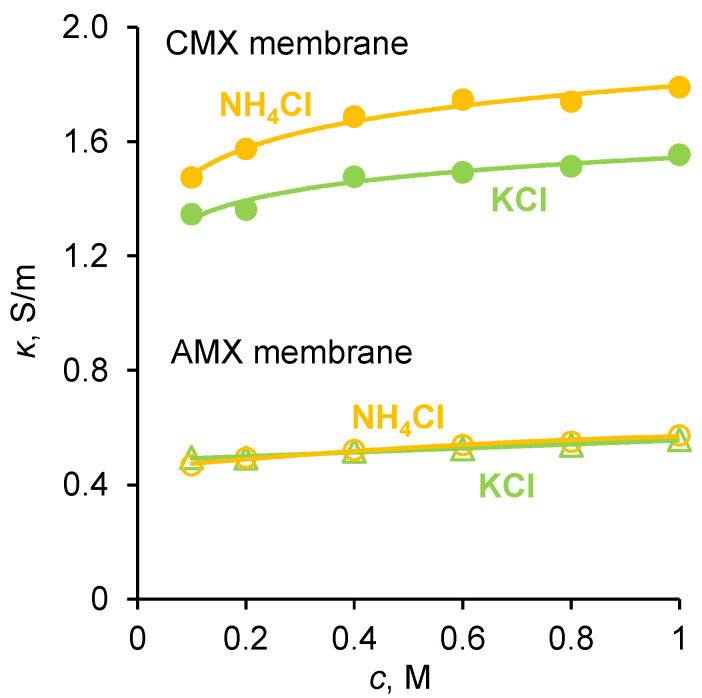
The conductivity of CMX and AMX membranes in KCl and NH_4_Cl solutions. The dots correspond to the experimental data; the solid lines are presented to lead the eye.

The membrane conductivity, *κ*, (in S/m) was measured as described in Section 3.3. The results of the measurements of *P* and *κ* of the membranes under study in KCl and NH_4_Cl solutions are presented in Figure 2 and Figure 3. 

According to theoretical and experimental studies [40,41,42,43], the diffusion permeability of AEMs and CEMs is controlled by the transport of coions, while the conductivity is controlled by the transport of counterions. Both quantities increase with increasing the solution concentration, since the concentrations of both counterions and coions in the membrane’s growth as the solution concentration, *c*, increases. Moreover, the coion concentration in the micropores increases approximately as *c*^2^ [40,44]. As Figure 2a shows, the diffusion permeability of the homogeneous Neosepta cation-exchange CMX membrane (Astom Corp., Takaoka, Japan, see more details about properties in Section 3.1) in KCl and NH_4_Cl solutions weakly depends on the type of electrolyte, since the coion, Cl^−^, is the same in both solutions. Similarly, the conductivity of the homogeneous Neosepta anion-exchange AMX membrane (Astom Corp., Japan) in both electrolytes is very close, since the counterion is the same.

Note also that the self-diffusion coefficients of K^+^ and NH_4_^+^ in solution have very close values (1.957 × 10^−9^ m^2^/s in an infinite dilute solution [28,45]). However, the conductivity of CMX in the NH_4_^+^ form is slightly (by about 15%) greater than that in the K^+^ form. Therefore, we can assume that the diffusion coefficient of NH_4_^+^ in this membrane is slightly greater than that of K^+^. This difference could be due to a slightly higher CMX membrane hydration in the presence of NH_4_^+^ compared to K^+^. The possible reason for this is due to the fact that, according to Hua et al. [46], NH_4_Cl perturbs water’s hydrogen-bonding network more significantly than KCl. Additionally, the experimental data of Fuoco et al. [47] show that the freezing point of water in the CMX membrane equilibrated with KCl solution is −15.9 °C, and with NH_4_Cl is −12.8 °C. These results allow us to conclude that, in the case of ammonium, the pores are larger and water is less bound, which explains the higher mobility of ammonium ions and the higher conductivity of the membrane in the form of these ions. As for the integral diffusion permeability coefficient of the AMX membrane, it is almost twice as much if the membrane contacts an NH_4_Cl solution, compared to a KCl solution (Figure 2b). The value of *P* is proportional to the ${\bar{D}}_{2}{\bar{c}}_{2}$ product, where ${\bar{D}}_{2}$ and ${\bar{c}}_{2}$ are the diffusion coefficient and concentration of coion (subscript 2), respectively [40,48]; see also Supplementary Materials. We do not observe the reasons why the diffusion coefficient and concentration of NH_4_^+^ ions would be much greater than those of K^+^ ions in an AMX membrane. Both cations, due to electrostatic repulsion from the positively charged quaternary ammonium groups (comprising most of the fixed functional groups of the AMX membrane), are not able to approach the fixed groups of the membrane matrix and interact with them. We are more inclined to accept the hypothesis expressed in the Introduction, which suggests that the elevated transfer of nitrogen through an AEM, such as AMX, is due to the contribution of ammonia molecules. These uncharged molecules can approach the fixed charged groups; hence, they can occupy more space in the membrane, so that their concentration can be significant. This hypothesis is also supported by publications [49,50] on the permeability of gas-separation membranes. According to these publications, the presence of ammonium salts in the membrane matrix can significantly increase its selectivity with respect to ammonia. This fact has been repeatedly confirmed, on the basis of which a patented method for gas separation was developed [51]. Study [49] shows that the most probable mechanism of high ammonia transport is due to its great sorption: ammonia dissolves in ammonium thiocyanate and diffuses across the membrane.

### 2.2. Mathematical Modeling of Diffusion Permeability of the AMX Membrane

**Figure 4:**
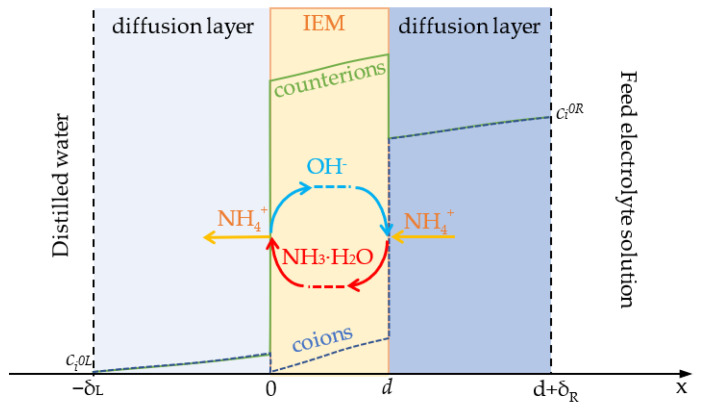
Schematic representation of the system under study. Here, *δ_L_*, *δ_R_* and *d* are the diffusion boundary layer (*L*—left hand and *R*—right hand) and membrane thicknesses, respectively.

We mathematically describe the following process. As mentioned in the Introduction, the pH of the internal solution in an AEM is higher than the pH of the external solution adjacent to the membrane surface, since H^+^ ions are expelled from the membrane as coions. Therefore, when NH_4_^+^ ions enter the membrane under the action of their concentration gradient, some of them are deprotonated and converted into NH_3_ molecules (Figure 4). The NH_3_ molecules diffuse through the membrane to its boundary with the depleted solution, which is initially distilled water. When leaving the membrane, they are protonated and again return to the form of NH_4_^+^ ions. The released OH^−^ ions return to the membrane boundary contacting with the feed solution. Here, these ions take part in the reaction of deprotonation of new NH_4_^+^ ions entering the membrane.

The 1D steady-state model of the diffusion transport of ammonium chloride through an AEM was developed. A three-layer system consisting of an AEM and two adjacent diffusion layers is considered (Figure 4). The membrane was placed between an NH_4_Cl solution and distilled water, and the diffusion of ions from the solution to the distilled water through the membrane was studied. The Nernst–Planck equations involving ion and molecule activity coefficients coupled with Poisson’s equation were applied. In the membrane, the transport of ions was modeled within the pores with charged walls, where the concentrations were considered as averaged over the pore cross-section. Since ions can only pass through the pores, the flux density per square meter of the membrane cross-section was found by multiplying the flux density through the pore (in mol s^−1^m^2^ pore cross-section) on the membrane porosity *p* (assumed equal to 0.3 for AMX, as typical value for membranes made by the paste method). However, as mentioned above, ammonia non-charged species can transfer not only inside the pores, but also inside the non-charged fragments of the membrane matrix. Therefore, the flux density found for the NH_3_ species was not multiplied by *p*. 

Ammonia protonation–deprotonation and water dissociation–recombination reactions were taken into account with finite rate constants. Within the solution/membrane interfaces (of the thickness of about 1 nm), we assumed the continuity of the activity of all species when passing through the interface between the solution and membrane; with that, the activity coefficients continuously changed from their values in the solution (where they were equal to 1) to their specific values in the membrane. Furthermore, the electric potential continuously changed in the interface. This assured the continuity of the electrochemical potential of each species in the interface. The mathematical formulation of the model is described in detail in Section 4 “Mathematical model”. The input parameters are discussed below; they are all presented in Table 1. Their determination is described in detail in the Supplementary Materials.

**Figure 5:**
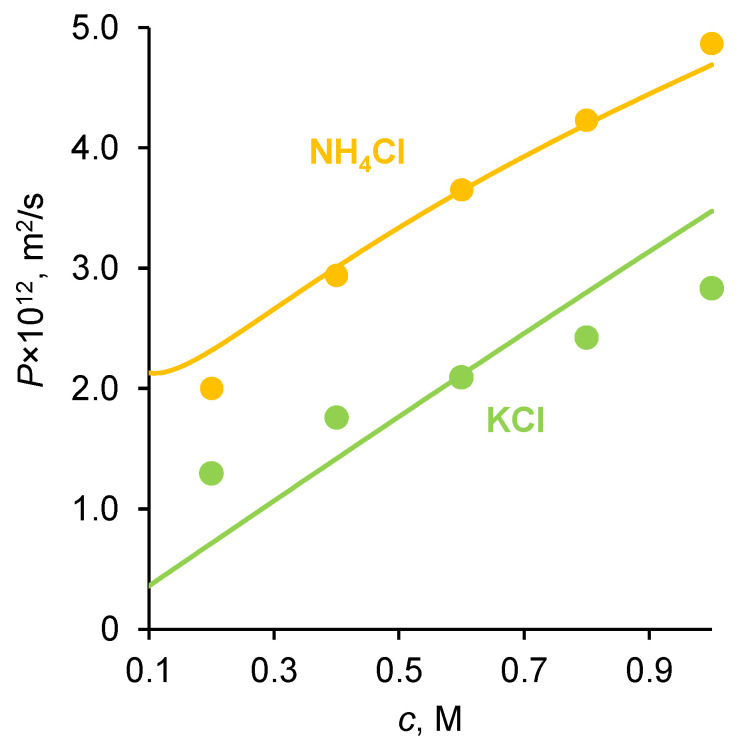
Dependence of the experimental (dots) and theoretical (lines) integral diffusion permeability coefficients of NH_4_Cl and KCl on the concentration of the external solution in the membrane system with the AMX membrane. The model parameters used in the calculation are presented in Table 1.

The processing of the experimental data and the adjustment of the activity coefficients made it possible to achieve good agreement between the theoretical and experimental data (Figure 5). 

The mathematical problem was numerically solved by the finite element method using the Comsol Multiphysics 5.6 commercial software package. 

### 2.3. Determination of the Input Parameters 

**Activity coefficients** in solution were the same and equal to 1 for all species. Activity coefficients in the membrane were selected, taking into account the affinity of the membrane for some specific species. As mentioned in Section 2.1, NH_3_ can be absorbed not only within the pores, but also within the membrane matrix not containing water [49]. The continuity activity condition at the interfaces used in the model assumes that for a species *i* (on the left side of the membrane):

(2) $$a_{i}\left(0-,t\right)=a_{i}\left(0+,t\right)$$

It follows from Equation (2) and the definition $a_{i}=c_{i}\gamma_{i}$ that

(3) $$c_{i}\left(0+,t\right)=c_{i}\left(0-,t\right)\gamma_{i}/{\bar{\gamma }}_{i}$$

where $\gamma_{i}/{\bar{\gamma }}_{i}=K_{s}$ is the partition coefficient, and the overbar means that the value refers to the membrane phase. We use the value ${\bar{\gamma }}_{{\mathrm{NH}}_{3}}$ = 0.03, which gives $K_{s}$ = 33 for ammonia molecules. Similarly, the value of ${\bar{\gamma }}_{{\mathrm{OH}}^{-}}$ is assumed to be 0.02, since it is known that the pH of the internal solution of the AMX membrane is quite elevated and reaches about 10–11, according to the measurements conducted by using a color indicator (anthocyanin), when the external solution is 0.02 M NH_4_Cl or KCl [22].

**Diffusion coefficients in the membrane.** The model of a homogeneous membrane used in this work (similar to the Teorell, Meyer and Sievers model [40]) describes, quantitatively, the properties of the membrane only in a small range of the external solution concentration (up to 0.2 M). To describe the properties of the system for a wider range of concentrations, effective diffusion coefficients, which depend on the concentration of the external solution, should be used. Theoretically, the dependence of effective diffusion coefficients on concentration can be taken into account if a model, which takes into account the heterogeneous structure of the membrane (for example, the microheterogeneous model [56]), is applied. However, the use of such a model would significantly increase the mathematical difficulties and complicate the understanding of the reasons for the high diffusion permeability of AEMs for NH_4_Cl. In this paper, of greatest interest is the concentration range (0.5–1.0 M), in which electrodialysis concentration or conversion of ammonium-containing solutions usually occurs. Therefore, we focused on this range of concentrations.

For the CMX membrane, the K^+^ and NH_4_^+^ ions are counterions; inside the membrane, they are electrostatically attracted by fixed groups, which, at a distance less than the Bjerrum length, leads to possible specific interactions [57]. Figure 3 shows that the electrical conductivity of CMX in KCl and NH_4_Cl solutions differs by no more than 15%. This means that the diffusion coefficients of K^+^ and NH_4_^+^ in the cation-exchange membrane can differ by no more than 15%. The calculations, according to Equation (S7) in the Supplementary Materials, produce the diffusion coefficients of K^+^ and NH_4_^+^ in the CMX membrane equal to 5.2 ± 0.2 × 10^−11^ m^2^/s and 6.0 ± 0.2 × 10^−11^ m^2^/s, respectively (at a feed solution concentration of 0.4–1 M). In the case of an anion-exchange membrane, the K^+^ and NH_4_^+^ ions are coions. Inside an AEM, fixed groups electrostatically repel them. Furthermore, the difference between the diffusion coefficients of K^+^ and NH_4_^+^ inside the anion-exchange membrane should not be large, since their diffusion coefficients are the same in a free solution. Based on the foregoing observations, we can assume that the diffusion coefficients of K^+^ and NH_4_^+^ in AEM are approximately the same. 

The calculations show that a change in the activity coefficients of H^+^ and OH^−^ ions in the range from 0.02 to 10 in the case of a KCl solution did not significantly affect the value of the KCl diffusion flux through the AEM. This flux at the 1 M feed solution concentration was 2.22 ± 0.02 × 10^−5^ mol/(m^2^s), which corresponds to *P* = 2.8 ± 0.4 × 10^−12^ m^2^/s, and was in a good agreement with experiment (Figure 5). The deviation in the calculated values of the flux when varying the values ${\bar{\gamma }}_{{\mathrm{OH}}^{-}}$ and ${\bar{\gamma }}_{H^{+}}$ did not exceed 0.1%. In other words, a change in the activity coefficients ${\bar{\gamma }}_{{\mathrm{OH}}^{-}}$ and ${\bar{\gamma }}_{H^{+}}$ should not lead to a change in the KCl diffusion flux through the membrane, since the presence/absence of OH^−^ and H^+^ ions does not affect the equilibrium of the potassium chloride dissociation reaction, and, as a result, its flux. In fact, KCl is a strong electrolyte: in the studied pH and concentrations ranges, it almost completely dissociates into K^+^ and Cl^–^ ions in aqueous solutions.

**Figure 6:**
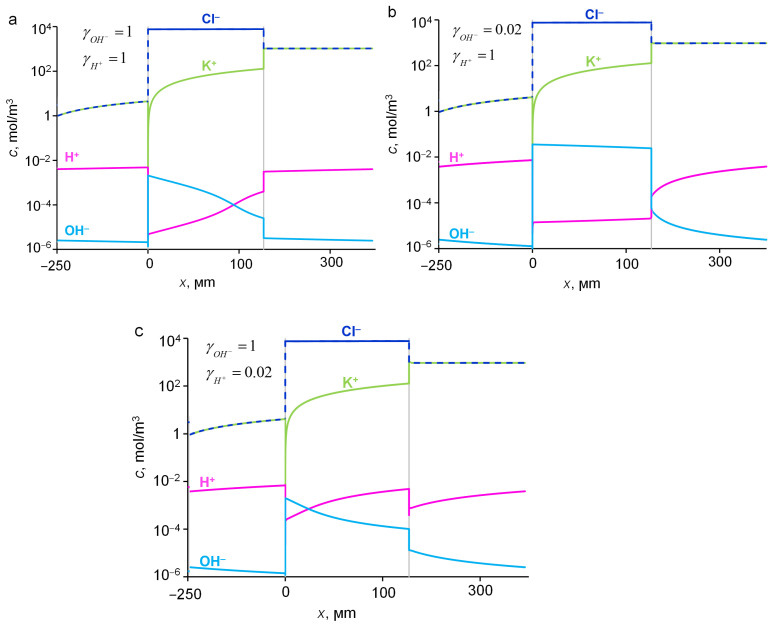
Distribution of ion concentrations in the system under study at different ${\bar{\gamma }}_{{\mathrm{OH}}^{-}}$ and ${\bar{\gamma }}_{H^{+}}$ (indicated in the plots) at 1 M of feed electrolyte KCl solution: 1 and 1 (**a**), 0.02 and 1 (**b**), 1 and 0.02 (**c**), respectively. Simulation with the input parameters presented in Table 1.

Figure 6 shows the concentration profiles of the components of an aqueous solution of KCl in AEM and in adjacent diffusion layers. Inside the membrane, at the boundary with the feed electrolyte solution, the concentrations of K^+^ and Cl^–^ ions take the maximum values: the concentration of counterions Cl^–^ is close to the ion-exchange capacity of AEM, and the concentration of coions is many times lower due to the Donnan (electrostatic) exclusion of coions. As it is known [40], this effect is enhanced with the dilution of the external solution. Therefore, at the side of the AOM adjoining the dilute solution (initially distilled water), an even more significant decrease in the concentration of coions (K^+^ and H^+^) in the membrane was observed, at least by three orders of magnitude compared to Cl^−^ anions. Due to the low concentration of H^+^ coions at the boundary with a dilute solution, the concentration of OH^–^ ions at this boundary reached its highest value in the membrane, which increased with decreasing ${\bar{\gamma }}_{{\mathrm{OH}}^{-}}$ and reached 2 × 10^−5^ M (pH = 9.9) at ${\bar{\gamma }}_{{\mathrm{OH}}^{-}}$ = 0.03.

The fact that the flux of KCl through an AEM does not depend on ${\bar{\gamma }}_{{\mathrm{OH}}^{-}}$, reduces the number of influencing parameters on the results of the simulation of KCl diffusion through the AMX membrane and allows fitting the diffusion coefficient of K^+^ in this membrane, which produces ${\bar{D}}_{K^{+}}$ = 2.7 × 10^−11^ m^2^/s. As follows from the above analysis, the diffusion coefficient of NH_4_^+^ in this membrane should be very close to that of K^+^. Therefore, we find ${\bar{D}}_{{\mathrm{NH}}_{4}^{+}}={\bar{D}}_{K^{+}}$ = 2.7 × 10^−11^ m^2^/s.

The diffusion coefficients of OH^−^, H^+^ and NH_3_ in the membrane were selected to be relatively high, only three times lower than the corresponding values in solution (Table 1), to match the high fluxes of NH_4_Cl through the AMX membrane, found experimentally. The fitting of ${\bar{\gamma }}_{{\mathrm{OH}}^{-}}$ and ${\bar{\gamma }}_{{\mathrm{NH}}_{3}}$ made it possible to achieve good agreement between the experimental and theoretical dependences of the diffusion permeability coefficient of the AMX membrane for NH_4_Cl. The best agreement was achieved with ${\bar{\gamma }}_{{\mathrm{OH}}^{-}}$ = 0.03 and ${\bar{\gamma }}_{{\mathrm{NH}}_{3}}$ = 0.03.

Further details of the determination of the diffusion coefficients in the membranes are presented in Section 4.2.

### 2.4. Concentrations and Fluxes in the Case of NH_4_Cl

**Figure 7:**
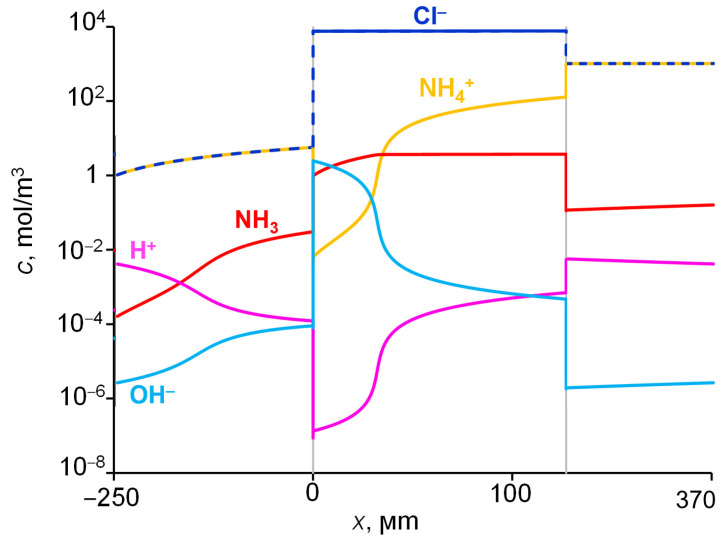
Distribution of ion concentration in the system under study at 1 M NH_4_Cl solution. Simulation with the input parameters presented in Table 1.

Figure 7 shows the distribution of the concentrations of all species present in the aqueous NH_4_Cl solution when ammonium chloride diffuses through an AEM from a feed solution to water. The calculations were made for the input parameters shown in Table 1. The concentration distribution of the products of the protonation–deprotonation reactions of ammonia species in the membrane was essentially determined by the local pH value. The shift of the pH in the membrane to the alkaline region lead to the transformation of a part of the NH_4_^+^ ions into neutral NH_3_ molecules. At the left-hand membrane boundary, the concentration of NH_3_ exceeds the concentration of NH_4_^+^ ions by more than two orders of magnitude. Simultaneously, the concentration profile of these species in the right-hand part of the membrane remains almost constant. However, the closer the membrane boundary to the dilute solution (initially distilled water), the higher the mole fraction of the molecular form in the couple NH_4_^+^/NH_3_. At the point where the pH value of the internal membrane solution reached 7.7, the concentrations of NH_4_^+^ and NH_3_ became equal, and at pH ≥ 8.8, the NH_4_^+^ concentration became smaller than the NH_3_ concentration. These pH values in the membrane were lower than the corresponding values in solution (see Figure 1), because, in the membrane, we used the activity coefficients, which were significantly less than 1 for NH_3_ and OH^−^; all activity coefficients in solution were equal to 1. 

**Figure 8:**
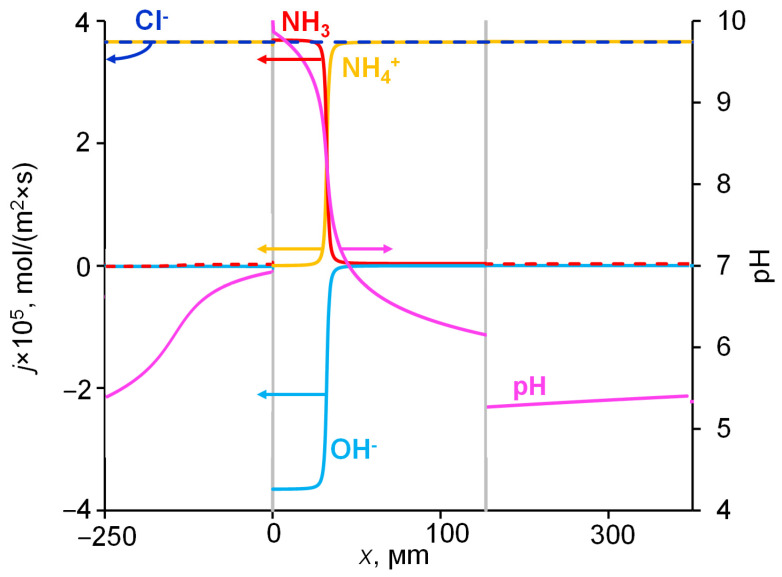
Dependence of the fluxes of all species present in the membrane system and pH dependence on the coordinate. The flux of H^+^ ions is negligibly small in all parts of the system and not shown. The secondary *y* axis refers to pH. Simulation for an AMX membrane and 1 M NH_4_Cl feed solution with the input parameters shown in Table 1.

The distribution of fluxes of diffusing species in the membrane system is shown in Figure 8. It can be observed that, when approaching the left-hand membrane boundary, a change in the nitrogen-atom carriers occurs, while the magnitude of the flux of these atoms does not change along the coordinate. The nitrogen transfer in the right half of the membrane is mainly performed by NH_4_^+^, and in the left half, mainly by NH_3_. The NH_3_ flux is negligible in the right-hand membrane part, while the NH_4_^+^ flux is negligible in the left-hand membrane part. When NH_3_ exits the membrane into the distillate, these molecules are protonated, and NH_4_^+^ ions are formed; thus, nitrogen enters the solution as part of the NH_4_^+^ ions. The released OH^–^ ions are transferred to the right-hand membrane boundary, these ions are then consumed in the reaction of NH_4_^+^ deprotonation in the membrane bulk. We assume that NH_3_ molecules can move not only through the membrane pores, but through the membrane matrix not containing water; in addition, the effective diffusion coefficient of NH_3_ in the membrane is taken greater than that of NH_4_^+^. For these reasons, the NH_3_ flux in the left-hand part of the membrane, which is equal to the NH_4_^+^ flux in the right-hand part (Figure 8), occurs at a lower concentration gradient of NH_3_ compared to that of NH_4_^+^ in the right-hand part of the membrane (Figure 7).

**Figure 9:**
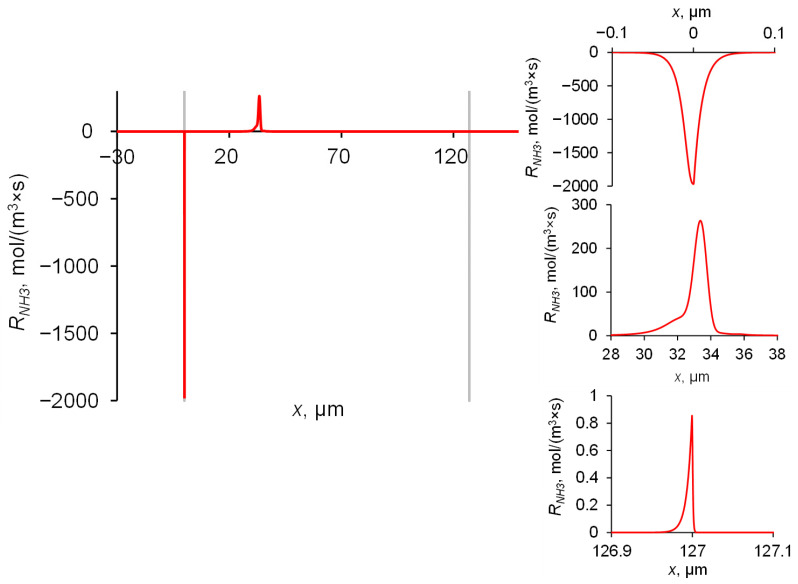
Dependence of the rate of NH_3_ molecules’ formations on the coordinate. Magnifications of three reaction zones are presented separately in appropriate scales. Simulation for an AMX membrane and 1 M NH_4_Cl feed solution with the input parameters shown in Table 1.

The rate of NH_3_ molecule formation as a function of the coordinate is shown in Figure 9. A small amount of this substance is formed in a narrow reaction zone (~0.03 μm thick) on the right-hand side of the membrane (*x* = 127 ± 5 μm), where NH_4_^+^ enters the membrane, in which the pH (6.2) is slightly higher than that in the boundary solution (5.3). The main amount of NH_3_ is generated in the reaction zone within the membrane bulk, in the vicinity of *x* = 33 μm; the thickness of this zone is ~4 μm. Here, two relatively high fluxes of NH_4_^+^ and OH^−^ ions meet moving towards each other. The third reaction region (~0.1 μm thick) is located on the left-hand side of the membrane, where NH_3_ molecules disappear to form NH_4_^+^ and OH^−^ ions. Here, an abrupt shoot of pH occurs when passing from the membrane (pH = 10) into solution (pH = 6.9). The largest reaction zone is formed in the membrane bulk, where two reactant fluxes gradually decrease in absolute value as they approach the point, at which both fluxes vanish, becoming much less than the flux of NH_3_ molecules. When the proton-exchange reactions occur in the interface between the two phases, the reaction zone is significantly smaller. 

### 2.5. Influence of pH of External Solution

**Figure 10:**
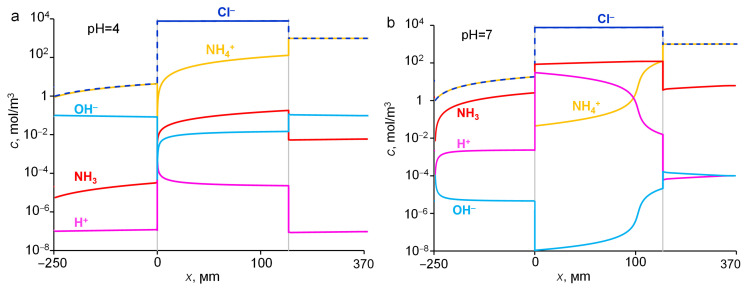
Distribution of species concentrations in the system under study at pH = 4 (**a**) and pH = 7 (**b**) of the 1 M NH_4_Cl feed solution. Simulation: the input parameters are presented in Table 1.

As it was mentioned above, the concentration of H^+^ and OH^−^ ions in the membrane directly depends on their concentration in the external solution. A change in the pH value of the external solution by 1.5 units leads to a dramatic change in the species concentrations in the membrane (compare Figure 7 and Figure 10). When passing from pH = 4 to pH = 7 in the feed solution, the concentration of NH_3_ at the right-hand side of the membrane increases by almost 3 orders of magnitude (Figure 10a,b).

**Figure 11:**
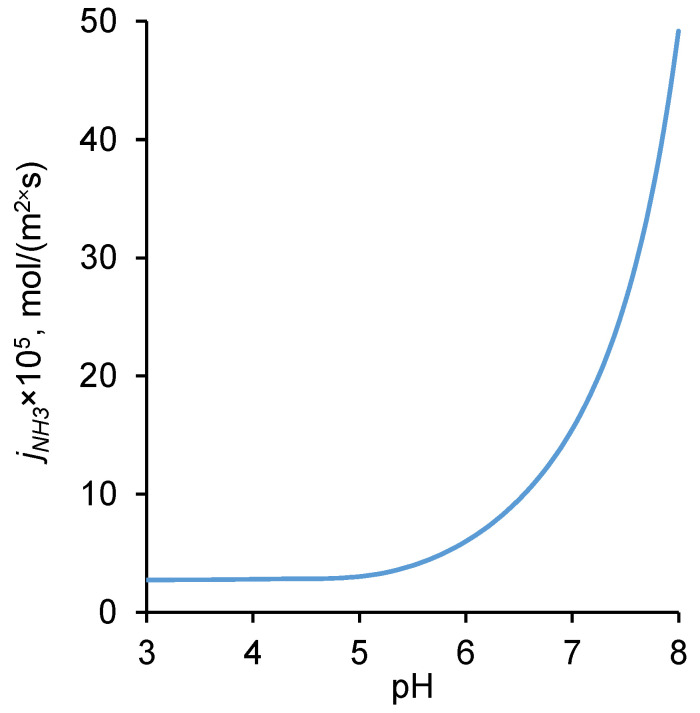
Dependence of the theoretical NH_4_Cl flux through an AMX membrane on the pH of the feed electrolyte solution. The input parameters are presented in Table 1.

As the pH of the external solution increases, the diffusion flux of NH_4_Cl increases (Figure 11). This is of practical importance, since a serious problem in the electrodialysis of ammonium-containing solutions is ammonium back diffusion [58]. Lowering the pH value by adding chemical reagents or creating a reagent-free system using a bipolar membrane would significantly reduce the parasitic ammonium flux. However, in the case of bipolar membranes, reducing the ammonium flux by controlling the pH of the external solution is not possible, since the concentration of H^+^ and OH^−^ ions in such membranes is determined by the rate of water splitting in the bipolar interfacial region [59], and not by the pH of the external solution, as in the case of a monopolar membrane. 

### 2.6. Discussion

The results of the simulation show that, generally, the mechanism of the enhanced permeability of an AEM with respect to NH_4_Cl diffusion, schematically depicted in Figure 4, is correct. Indeed, NH_4_^+^ ions react in the membrane with OH^−^ ions, thus turning into NH_3_ molecules. OH^−^ ions are generated at the membrane interface facing distilled water when NH_3_ molecules leave the membrane and enter the water. However, there is an important detail: the conversion of NH_4_^+^ into NH_3_ occur not only in the membrane interface facing the feed solution. The degree of conversion gradually increases with the increasing distance from the membrane side facing the feed solution and approaching the side facing the dilute solution; that is, as far as the pH of the internal solution rises (Figure 7 and Figure 8). All the membrane volumes may be divided into three parts. In the right-hand part, the nitrogen atoms are principally transported by NH_4_^+^ ions and the flux of NH_3_ molecules is negligible; here, the value of the pH changes from 6.2 to 7.5. In the left-hand part, the nitrogen is mainly transported by NH_3_ and the flux of NH_4_^+^ ions is negligible; here, the pH changes from 8.8 to 10.0 (Figure 8). There is a narrow central part, where the change of the nitrogen-atom carrier occurs. Within this layer, the fluxes of NH_4_^+^ and NH_3_ are comparable; when passing from the left to the right, the flux of OH^−^ ions abruptly decreases in absolute value from the value equal to the NH_3_ flux at the left side to almost zero at the right side of the membrane. The OH^−^ ions moving from left to right react with NH_4_^+^ ions moving in the opposite direction.

Note that the NH_4_^+^ concentration reaches almost zero at a point close to the middle of the membrane. Therefore, the effective thickness of the diffusion layer for the transport of this ion decreases by about a factor of two, when compared to the case when the conversion of NH_4_^+^ ions into NO_3_ molecules does not occur. When NO_3_ takes over the nitrogen transport, it only needs to pass through the other half of the membrane. Thus, the flux of nitrogen through an anion-exchange membrane during NH_4_Cl diffusion can be doubled, when compared to the transport of another atom (such as potassium), which is carrried by an ion (K^+^) with the same mobility as NH_4_^+^, but cannot be converted into a neutral species during KCl diffusion through the same membrane. 

Since the rate constant of this reaction (*k*_−1_, Equation (9), Section 4.1) was high, the reaction was limited by the values of the fluxes of OH^−^ and NH_4_^+^. OH^−^ ions were generated on the left side of the membrane, when NH_3_ molecules left the membrane and entered a medium with a relatively low pH, where reaction (9) (Section 4.1) occurred. The NH_4_^+^ ions formed in this reaction transferred from the membrane/solution interface towards the bulk of initially distilled water, and the other product, OH^−^ ions, moved towards the right side of the membrane. The rate constant of this reaction (*k*_1_, Equation (9)) was also very high. Note that all the rate constants involved in reactions (9)–(11) were high, except for only the water-dissociation rate constant, *k_d_*, reaction (11). However, this could not slow down the resulting rate generation of H^+^ and OH^−^ ions, since they could be obtained in reactions (9) and (10). On the contrary, it can be argued (and this was confirmed in experiment [19,22]) that the presence of ammonium in a solution subjected to electrodialysis leads to an increase in the rate of H^+^/OH^−^ ion generation near an AEM surface when a sufficiently high-current density flows through the membrane. These ions are not formed as a result of water dissociation, reaction (11), but as a result of reactions (9) and (10) of the protonation–deprotonation reaction of NH_3_ and NH_4_^+^ particles, respectively. A similar H^+^/OH^−^ ion-generation mechanism was described by Simons [52,60] and other authors [61] for substances, presented in the solution near an IEM, which can be involved in similar proton-exchange reactions. Since the substances, such as NH_3_ and NH_4_^+^, are not used up in this process, and only water molecules are consumed, this process is known as “water splitting”. The possibility of this effect occurring in biological membranes is discussed in the literature [60]. 

It follows from the foregoing that, in the system under consideration, there were no kinetic limitations on the part of the chemical reactions. Therefore, we could reduce the model by assuming local chemical equilibrium not only at the interfaces (Equations (17) and (18)), but at any point in the system, and therefore used these equations everywhere instead of Equations (12), (13), (15) and (16). However, the use of Equations (12)–(16) only slightly complicated the numerical solution of the mathematical problem. On the other hand, such use made the model more general and applicable not only at zero electric current, but even at relatively high-current densities when some reactions could be kinetically limiting. 

The use of Poisson’s equation instead of the simpler local electroneutrality condition can be characterized in a manner similar to that described above: the model with Poisson’s equation can not only be applied in the conditions of electrolyte diffusion, but also under electric current flow. There is another advantage of the application of Poisson’s equation. When using the local electroneutrality condition, for some input parameters, ion concentrations at the interfaces become so small that negative concentrations can appear during the numerical solution process. In this case, the program crashes. When the Poisson’s equation is applied, this difficulty does not occur.

Note that similar processes can occur in bipolar membranes (BPMs) during their use in the electrodialysis of ammonium-containing solution, which explain a very high permeation of ammonia through these membranes known in the literature [25,26,58]. The difference is that the OH^−^ ions in BPMs are mainly formed in the bipolar interfacial region, where water splitting is enhanced by the catalytic participation of fixed functional groups. A high pH value of the internal solution of the anion-exchange layer causes a high concentration of ammonia in this layer, which is the main carrier of the nitrogen atom in it. In the cation-exchange layer, the nitrogen atom is carried by NH_4_^+^ cations due to a very low pH value in this layer. 

Note that artificial IEM and biological membranes are similar to each other: the design of both provides selectivity with respect to a certain type of ions by the formation of channels/pores that have a specific permeability only for this type of ion. Moreover, the ingenuity of nature, as in many other cases, surpasses that of man: biological membranes have a higher selectivity than synthetic ones. However, with regard to the enhanced transport of ammonium studied here, in the case of biological membranes, there is also evidence of the undesirable penetration of ammonium ions through a cell membrane that was not intended to be permeable to them [29,30,31]. The general view of the causes of such elevated NH_4_^+^ transport through cell membranes is non-ionic diffusion [62,63]. According to this explanation, the ionized form of the compound transforms into its non-ionized configuration upon crossing the membrane surface, which can subsequently diffuse through the nonpolar region of the cell membrane [63,64]. The model presented in this paper describes such a mechanism in detail from a physicochemical point of view. We believe that this model can not only be useful for specialists in artificial membranes, but also for those who study the selective transport of nitrogen through cell membranes. 

## 3. Experimental Part

### 3.1. Membranes and Solutions

Homogeneous ion-exchange membranes Neosepta CMX and AMX (Astom Corp., Japan) manufactured by the paste method [65] were used in the study. Both membranes consisted of a randomly cross-linked functionated styrenedivinylbenzene copolymer (45–65%) and polyvinylchloride (45–55%), and were reinforced with a polyvinyl chloride mesh. The CMX is a cation-exchange membrane and contains fixed sulfonic groups; the AMX is an anion-exchange membrane and contains quaternary ammonium bases and a small amount of secondary and tertiary amines [66]. 

The solutions of KCl and NH_4_Cl were prepared from a crystalline salt (analytical grade) provided by OJSC Vekton (Yekaterinburg, Russia); the 0.10 M KOH solution was prepared from a titrant (manufactured by Uralkhiminvest, Ufa, Russia). KOH was used to maintain a constant pH value of the solution circulating through the compartments. Distilled water of electrical conductivity 0.8 μS cm^−1^ and pH = 6.2 ± 0.2 at 25 °C was used to prepare the solutions.

All membrane samples underwent a standard salt pretreatment [67] and were then equilibrated with 0.02 M KCl or 0.02 M NH_4_Cl solutions before experiments.

### 3.2. Diffusion Permeability

**Figure 12:**
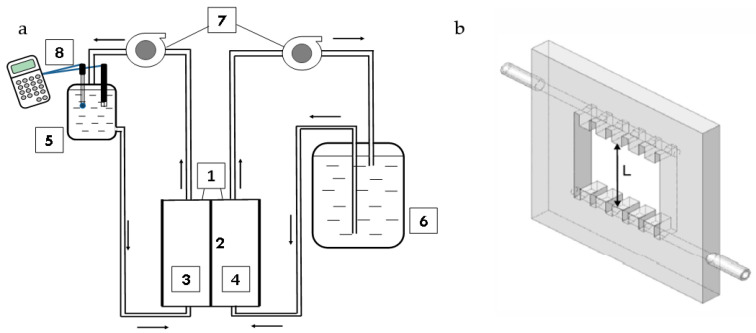
(**a**) Schematic representation of the experimental setup for measuring the membrane diffusion permeability: (1) two-compartment cell, (2) membrane under study confined between two frames shown in (**b**), (3, 4) flow-through compartments of cell (1), (5) container with initially distilled water, (6) container with the electrolyte solution of a given concentration, (7) pumps, and (8) conductometric and pH electrodes connected with conductometer and pH meter, respectively; (**b**) plastic frame with special comb-shaped guides of liquid flow.

The diffusion characteristics of IEMs were investigated using an experimental setup schematically represented in Figure 12. The two-compartment flow cell was formed by solid plastic frames (1) and a membrane (2), the active surface of which was equal to 7.3 cm^2^. The distance between the surfaces of the membrane (2) and the solid plastic frame forming the external wall of the cell was 6.3 mm. The plastic frames with a square aperture were equipped with special comb-shaped guides, which provided the laminar regime of the solution flow in the cell compartments. The membrane separated two streams: distilled water was pumped through one of them (stream I), and a NH_4_Cl or KCl solution of a given concentration and pH was pumped through the other stream (II). Before the experiments, all samples were equilibrated with 0.02 M solution of considered electrolyte (NH_4_Cl or KCl). The first measurements were performed for the concentration of the electrolyte solution in stream II (Figure 1) equal to 0.02 M. Then, this concentration was sequentially increased to 1.0 M. The membrane under investigation was in contact with each of the solutions for at least 5 h. The cell scheme, the methodology for conducting the experiment and processing the obtained data are described in detail in [68]. The confidence interval for determining the integral diffusion permeability coefficient of membranes for a given diffusion layer thickness was equal to ±0.4 × 10^−8^.

The integral diffusion coefficient is calculated using the following Equation (4):

(4) $$\;P=\frac{V_{dw}d}{Sc}\frac{dc_{dw}}{dt}$$

where $\frac{Vdc_{dw}}{Sdt}$ is the flux density of electrolyte diffusion through the membrane, $V_{dw}$ is the volume of the initially distilled water (filling container 5), *c_dw_* is the electrolyte concentration in the initially distilled water, *S* is the membrane area, *t* is time and *c* is the concentration of the feed solution (does not change during an experiment). 

### 3.3. Conductivity of IEM

The conductivity of IEM (*κ*) was determined by a differential method using a clip cell [69,70] and an immittance meter MOTECH MT4080 (Motech Industries Inc., Tainan, Taiwan) at an alternating current frequency of 1 kHz. All the samples were studied in 0.02 M–1.0 M solutions, starting from the lowest concentration.

The conductivity of the membranes (*κ*) is calculated using Equation (5):

(5) $$\;\kappa =\frac{d}{R_{m+s}-R_{s}}$$

where *R*_*m*+*s*_ is the resistance of the membrane and solution; *R_s_* is the resistance of the solution.

## 4. Mathematical Model

### 4.1. Model Formulation 

The system under study consisted of an anion-exchange membrane of thickness *d* with two adjacent diffusion layers (DLs): one faced the distilled water on the left side (thickness *δ_L_*) and the other faced the feed solution on the right side (thickness *δ_R_*), as shown in Figure 4. The thicknesses of both diffusion layers were calculated by the Leveque equation (Equation S1, Supplementary Materials). The transport of five species was considered: ammonium ions (NH_4_^+^), ammonia molecules (NH_3_), chloride ions (Cl^−^), hydrogen ions (H^+^) and hydroxyl ions (OH^−^). The transport of the species in the solution and membrane is described by the Nernst–Planck (6), Poisson’s Equation (7) and material-balance (8) equation system:

(6) $$j_{i}=-pD_{i}\left(\left(1+\frac{d\ln\gamma_{i}}{d\ln c_{i}}\right)\frac{\partial c_{i}}{\partial x}+z_{i}c_{i}\frac{F}{RT}\frac{d\varphi }{dx}\right)$$

(7) $$-\varepsilon_{r}\varepsilon_{0}\frac{\partial^{2}\varphi }{\partial x^{2}}=F\left(\sum_{i=1}^{n}z_{i}c_{i}+z_{m}Q\right)$$

(8) $$\frac{\partial j_{i}}{\partial x}=pR_{i}$$

where *j_i_*, *D_i_*, *z_i_*, *c_i_*, *γ_i_* and *R_i_* are the flux density, diffusion coefficient, charge value, concentration, activity coefficient and rate of generation of the *i*-th species (listed above), respectively; *x* is the space coordinate; *F* is the Faraday constant; *R* is the gas constant; *T* is the temperature; *φ* is the electric potential; *ε_r_* is the relative permittivity of the medium; *ε*_0_ is the vacuum permittivity; *z_m_* is the charge value of membrane fixed groups; *Q* is the concentration of fixed ions; *t* is time.

It was assumed that the ions mentioned above could transfer through the membrane only inside the pores; therefore, the Nernst–Planck equation in the membrane for these ions was written for the pore solution: the concentrations of mobile and fixed ions were taken in mole/m^3^ of the pore solution. To convert the ion-flux density found in mole s^−1^/m^2^ of the pore cross-section into the unit appropriate for coupling with the flux density in solution, i.e., mole s^−1^/m^2^ of membrane cross-section, we used coefficient *p*, which was the membrane porosity *p* (assumed equal to 0.3 for AMX, as a typical value for membranes made by the paste method [55,71]). Evidently, *p* = 1, when considering the transport in solution. As mentioned above, NH_3_ molecules can not only pass through pores, but also through uncharged fragments of the membrane matrix. Therefore, to calculate the flow of these species in the membrane, we also assume *p* = 1.

To find *R_i_*, three chemical reactions between the species are considered:

(9) $${\mathrm{NH}}_{3}+H_{2}O\underset{k_{-1}}{\overset{k_{1}}{⇄}}{\mathrm{NH}}_{4}^{+}+{\mathrm{OH}}^{-}$$

(10) $${\mathrm{NH}}_{4}^{+}\underset{k_{-2}}{\overset{k_{2}}{⇄}}{\mathrm{NH}}_{3}+H^{+}$$

(11) $$H_{2}O\underset{k_{r}}{\overset{k_{D}}{⇄}}H^{+}+{\mathrm{OH}}^{-}$$

The rates of generation for each species according to Equations (9)–(11) are as follows:

(12) $$R_{{\mathrm{NH}}_{3}}=-k_{1}a_{{\mathrm{NH}}_{3}}+k_{-1}a_{{\mathrm{NH}}_{4}^{+}}a_{{\mathrm{OH}}^{-}}+k_{2}a_{{\mathrm{NH}}_{4}^{+}}-k_{-2}a_{{\mathrm{NH}}_{3}}a_{H^{+}}$$

(13) $$R_{{\mathrm{NH}}_{4}^{+}}=k_{1}a_{{\mathrm{NH}}_{3}}-k_{-1}a_{{\mathrm{NH}}_{4}^{+}}a_{{\mathrm{OH}}^{-}}-k_{2}a_{{\mathrm{NH}}_{4}^{+}}+k_{-2}a_{{\mathrm{NH}}_{3}}a_{H^{+}}$$

(14) $$R_{{\mathrm{Cl}}^{-}}=0$$

(15) $$R_{H^{+}}=k_{2}a_{{\mathrm{NH}}_{4}^{+}}-k_{-2}a_{{\mathrm{NH}}_{3}}a_{H^{+}}+k_{d}a_{H_{2}O}-k_{r}a_{H^{+}}a_{{\mathrm{OH}}^{-}}$$

(16) $$R_{{\mathrm{OH}}^{-}}=k_{1}a_{{\mathrm{NH}}_{3}}-k_{-1}a_{{\mathrm{NH}}_{4}^{+}}a_{{\mathrm{OH}}^{-}}+k_{d}a_{H_{2}O}-k_{r}a_{H^{+}}a_{{\mathrm{OH}}^{-}}$$

where *a_i_* is the activity of *i*-th species; *k_i_* are the rate constants of protonation–deprotonation reactions presented in Equations (9)–(11).

### 4.2. Boundary Conditions

Equations (6)–(8) are valid for both DLs and the membrane. However, the values of the diffusion coefficients, activity coefficients, porosity, concentration of the fixed ions and relative permittivity in DLs and the membrane were different. These parameters smoothly varied at the membrane/solution interface (i.e., at *x* = 0 and *x* = *d*) from the values in the solution to those in the membrane. The thickness of the interface transition regions was chosen to be 1 nm, which was close to the value of the dense part of the double electrical layer [72]. To describe these changes, the rectangle function (in Comsol software, Stockholm, Sweden) was used. It was verified that a small variation in the transition region thickness (in the range from 1 to 2 nm) and the shape of the function describing the variation of the parameters did not affect the results of the numerical solution. 

It was assumed that at *x* = 0 and *x* = *d* there was a local equilibrium of reactions (9)–(11), which is described by the following equations:

(17) $$K_{b}=\frac{k_{1}}{k_{-1}}=\frac{a_{{\mathrm{NH}}_{4}^{+}}a_{{\mathrm{OH}}^{-}}}{a_{{\mathrm{NH}}_{3}}},\;K_{a}=\frac{k_{2}}{k_{-2}}=\frac{a_{{\mathrm{NH}}_{3}}a_{H^{+}}}{a_{{\mathrm{NH}}_{4}^{+}}}$$

(18) $$K_{w}=\frac{k_{d}}{k_{r}}=a_{H^{+}}a_{{\mathrm{OH}}^{-}}$$

where *K_b_* is the base ionization constant of ammonia; *K_w_* is the water-dissociation constant; *k*_1_ and *k*_−1_ are the rate constants of forward and reverse reactions (9), respectively; *k*_2_ and *k*_2_ are the rate constants of forward and reverse reactions (10), respectively; *k_d_* is the rate constant of water dissociation; *k_r_* is the rate constant of recombination H^+^ and OH^−^.

The concentrations of all species at *x* = −*δ_L_* were zero, except for concentrations of H^+^ and OH^−^ since there was distilled water in the bulk of the left-hand solution with pH = 5.4 ± 0.2; at *x* = *d* + *δ_R_* the concentrations, $c_{i}^{0R}$, are known from the experimental conditions:

(19) $$c_{i}\left(x=-\delta_{L}\right)=0,\;i={\mathrm{NH}}_{3},{\;\mathrm{NH}}_{4}^{+},{\;K}^{+},{\;\mathrm{Cl}}^{-};\;c_{H^{+}}\left(x=-\delta_{L}\right)=10^{-5.4};\;c_{{\mathrm{OH}}^{-}}\left(x=-\delta_{L}\right)=10^{-8.6}$$

(20) $$c_{i}\left(x=d+\delta_{R}\right)=c_{i}^{0R}$$

The concentration of chloride ions and pH value in the feed solution are set for each experimental run. Then, the concentrations of hydrogen and hydroxyl ions can be calculated from the known pH. The concentration of ammonium ions can be calculated using the electroneutrality assumption: $c_{{\mathrm{NH}}_{4}^{+}}=c_{{\mathrm{Cl}}^{-}}+c_{{\mathrm{OH}}^{-}}-c_{H^{+}}$.

The concentration of ammonia is calculated from Equation (17), the activity coefficients are set equal to 1:

(21) $$c_{{\mathrm{NH}}_{3}}^{0}=\frac{K_{a}\cdot c_{{\mathrm{NH}}_{4}^{+}}^{0}}{c_{H^{+}}^{0}}.$$

At *x* = −*δ_L_*, the electrical potential equals zero:

(22) $$\varphi \left(x=-\delta_{L}\right)=0$$

At *x* = −*δ_R_* and *x* = −*δ_L_*, the current density, *j*, equals zero:

(23) $$j=F\underset{i}{\sum }J_{i}z_{i}=0$$

## 5. Conclusions

We proposed a new one-dimensional model to explain the enhanced diffusion of ammonium chloride through anion-exchange membranes. The diffusion and migration transport of ions as well as proton-exchange reactions between NH_4_Cl, NH_3_ and water were taken into account. It was assumed that NH_4_^+^, Cl^−^ and OH^−^ ions could only pass through the hydrophilic pores of an AEM, while NH_3_ could diffuse both through the pores and through fragments of the membrane matrix that did not contain water. Another reason for a high NH_4_Cl diffusion was a lower value of the effective diffusion layer, *δ*_ef_, which controlled the rate of diffusion. In the case of KCl diffusion through the same membrane, *δ*_ef_ was equal to the membrane thickness; while, in the case of NH_4_Cl diffusion, it was approximately two-times lower. The latter was caused by the fact that the concentration of NH_4_^+^ almost reached zero value close to the middle of the membrane. In the other half of the membrane, the nitrogen atom was carried by NH_3_ molecules. These molecules formed in the membrane due to the reaction between NH_4_^+^ and OH^−^ ions. The latter were generated at the membrane interface facing distilled water when NH_3_ molecules left the membrane and entered the water. 

Overall, the model correctly described the concentration dependence of the diffusion permeability and electrical conductivity of an anion-exchange membrane (a Neosepta AMX membrane) in NH_4_Cl and KCl solutions. However, the calculated dependence was steeper than the experimental one. The reason is that the model did not take into account the contribution of ion transfer in an electrically neutral solution that fills the macropores and central parts of the mesopores of the membrane. This contribution can be taken into account when combining the proposed model and the known micro-heterogeneous model. However, at this stage of our study, this combination seems to be difficult to realize. We plan to create it in the future.

The model also provides an insight into the understanding of the high permeability of ammonia through bipolar membranes during the electrodialysis of an ammonium-containing solution. The difference is that the OH^−^ ions are mainly formed at the bipolar interfacial region, where water splitting enhanced by the catalytic participation of fixed functional groups occurs. A high pH value of the internal solution of the anion-exchange layer caused a high concentration of ammonia in this layer, which was the main carrier of the nitrogen atom. In the cation-exchange layer, the nitrogen atom was carried by NH_4_^+^ cations due to a very low pH value in this layer. 

We also believe that the model can be useful for achieving a better understanding of nitrogen transport across biological cell membranes.

## Figures and Tables

**Table 1:** Input parameters of the model for the system under study.

Parameter	Description	Value	Reference
*d*	Membrane thickness	AMX 127 ± 5 μm	*
		CMX 172 ± 5 μm	*
*δ_L_* = *δ_R_*	Diffusion-layer thickness	247 μm	Equation (S1)
	KCl (NH_4_Cl) electrolyte-diffusion coefficient in solution	1.99 × 10^−9^ m^2^/s	
*K_a_*	Acid-dissociation constant of NH_3_	5.62 × 10^−7^ mol/m^3^	[28]
*K_w_*	Water-dissociation constant	10^−8^ mol^2^/m^6^	[28]
*k*_1_	Rate constant of forward reaction (3)	1.78 × 10^5^ s^−1^	Equation (S2)
*k*_−1_	Rate constant of backward reaction (3)	10^7^ m^3^/(s × mol)	*k*_1_ × *K_b_*
*k*_2_	Rate constant of forward reaction (4)	5.63 s^−1^	Equation (S2)
*k*_−2_	Rate constant of backward reaction (4)	10^7^ m^3^/(s × mol)	*k*_2_ × *K_a_*
*k_d_*	Rate constant of water dissociation	2 × 10^−5^ s^−1^	[52]
*k_r_*	Rate constant of water recombination	1.18 × 10^8^ m^3^/(s × mol)	*k_d_*/(*K_w_* × *c_w_*)
*c_w_*	Concentration of water	5.55 × 10^4^ mol/m^3^	
$D_{{\mathrm{NH}}_{3}}$	Diffusion coefficients of species in solutions	1.64 × 10^−9^ m^2^/s	[53]
$D_{{\mathrm{NH}}_{4}^{+}}$		1.96 × 10^−9^ m^2^/s	[28]
$D_{{\mathrm{Cl}}^{-}}$		2.03 × 10^−9^ m^2^/s	[28]
$D_{H^{+}}$		9.3 × 10^−9^ m^2^/s	[28]
$D_{{\mathrm{OH}}^{-}}$		5.3 × 10^−9^ m^2^/s	[28]
$D_{K^{+}}$		1.96 × 10^−9^ m^2^/s	[28]
${\bar{D}}_{{\mathrm{NH}}_{3}}$	Diffusion coefficient of species in the membrane	4.4 × 10^−10^ m^2^/s	
${\bar{D}}_{{\mathrm{NH}}_{4}^{+}}$		2.7 × 10^−11^ m^2^/s	**
${\bar{D}}_{{\mathrm{Cl}}^{-}}$		2.7 × 10^−11^ m^2^/s	**
${\bar{D}}_{H^{+}}$		2.8 × 10^−9^ m^2^/s	
${\bar{D}}_{{\mathrm{OH}}^{-}}$		1.6 × 10^−9^ m^2^/s	
${\bar{D}}_{K^{+}}$		2.7 × 10^−11^ m^2^/s	**
$\gamma_{{\mathrm{NH}}_{3}}$	Activity coefficients of species in membrane	0.03	**
$\gamma_{{\mathrm{NH}}_{4}^{+}}$		1	
$\gamma_{{\mathrm{Cl}}^{-}}$		1	
$\gamma_{H^{+}}$		1	**
$\gamma_{{\mathrm{OH}}^{-}}$		0.03	**
$\gamma_{K^{+}}$		1	
*ε_s_*	Relative permittivity in solution	80	[28]
*ε_m_*	Relative permittivity in membrane	30	[54]
*Q*	Ion-exchange capacity	7600 ± 1000 mol/m^3^ H_2_O	[55]
pH	pH value in both streams	5.4 ± 0.2	*
$c_{{\mathrm{Cl}}^{-}}^{0R}$	Chloride-ion concentration at *x* = *d* + *δ_R_*	0.1–1 ± 0.001 M	*
*p*	Porosity	0.3	

*—parameters are from an independent experiment; **—fitting parameters.

## Data Availability

Not applicable.

## Supplementary Materials

The following supporting information can be downloaded at: https://www.mdpi.com/article/10.3390/ijms23105782/s1, Equations (S1)–(S8). Reference [73] is cited in the supplementary materials.
