# Supplementary material for: High Diffusion Permeability of Anion-Exchange Membranes for Ammonium Chloride: Experiment and Modeling
Source: Int J Mol Sci. 2022 May 21;23(10):5782. doi: 10.3390/ijms23105782 (PMC9147341; doi:10.3390/ijms23105782)
Supplement: Supplementary file 1 [file ijms-23-05782-s001.zip › ijms-1734551-supplementary.pdf]

High Diffusion Permeability of Anion Exchange Membranes for Ammonium Chloride: Experiment and Modeling

Ekaterina Skolotneva, Ksenia Tsygurina, Semyon Mareev, Ekaterina Melnikova, Natalia Pismenskaya and Victor Nikonenko*

Membrane Institute, Kuban State University, 149 Stavropolskaya st., 350040 Krasnodar, Russia; mareev-semyon@bk.ru (S.M.); kseniya_alx@mail.ru (K, T.); ek.skolotneva@gmail.com (E.S.); n_pismen@mail.ru (N. P.)

* Correspondence: v_nikonenko@mail.ru; Tel.: +7-918-414-5816

Supplementary materials

Determination of input parameters

The diffusion boundary layer thickness in the experimental cell is calculated using the Leveque equation [1]

$$\delta = 1.02\left(\frac{LDh}{\bar{V}}\right)^{1/3} \tag{S1}$$

here L is the channel length, D is the electrolyte diffusion coefficient, h is the distance between the studied membrane and the cell wall, $\bar{V}$ is the linear flow velocity.

Reaction rate constants. According to Simons [2], the rate constants k_{-1} and k_{-2} of recombination reactions (9) and (10) (such numbering hereinafter refers to the equations from the main text of the article) free solution have high values of the order of 10^{10} dm^3 mol^{-1} s^{-1}. The rate constants k_1 and k_2 of direct reactions (9) and (10) (considered as pseudomonomolecular) can be estimated using the following relations [23]:

$$k_1 = k_{-1}10^{-(14-pK_a)},\ k_2 = k_{-2}10^{-pK_a} \tag{S2}$$

The calculation using equation (S2) gives $k_1 \approx 10^6\ s^{-1}$, $k_2 \approx 10\ s^{-1}$. Thus, the rate limiting stage of protonation-deprotonation reactions (9) and (10) is the NH_4^+ deprotonation reaction. However, the value of the rate constant of this reaction (k_2) is quite high. It is almost 6 orders of magnitude higher than the rate constant of water dissociation in free solution (2×10^{-5} s^1) , 4 orders of magnitude higher than in the case of sulfonic groups, 3×10^{-3} s^{-1} [3] (the CMX membrane) and 2 orders of magnitude higher than in the case of secondary and tertiary amino groups, 10^{-1} s^{-1} [3] (the AMX membrane). Thus, NH_4^+/NH_3 couple may be considered as a catalyst for the reaction of H^+ and OH^- ions generation. The latter is important for electrodialysis of ammonium-containing solutions, where water splitting reactions are of great impact.

Diffusion coefficients. The model assumes that the membrane is a quasi-homogeneous medium. The diffusion coefficients may be estimated from the equation system, describing the conductivity, κ, and differential diffusion permeability, $\bar{P}$, of the membrane [4]:

$$\bar{P} = \bar{t}_1\left(1-\frac{z_2}{z_1}\right)\frac{\bar{D}_2\bar{c}_2}{c_2} \tag{S3}$$

$$\kappa = \frac{F^2}{RT}\left(z_1^2\bar{D}_1\bar{c}_1 + z_2^2\bar{D}_2\bar{c}_2\right) \tag{S4}$$

$$\bar{t}_i = \frac{z_i^2 \bar{D}_i \bar{c}_i}{z_1^2 \bar{D}_1 \bar{c}_1 + z_2^2 \bar{D}_2 \bar{c}_2} \quad \text{(S5)}$$

where subscript 1 and 2 refer to the counterion and coion, respectively, $\bar{t}_i$ is the transport number of ion *i* in the membrane.

Since $\bar{t}_1$ is close to 1, it can be seen from Equation (S3) that $\bar{P}$ is mainly determined by the product $\bar{D}_2\bar{c}_2$ at a given concentration of the external solution, c_2. As $\bar{D}_2\bar{c}_2 << \bar{D}_1\bar{c}_1$, the membrane conductivity is controlled by the product $\bar{D}_1\bar{c}_1$, Equation (S4). At a given concentration of the external solution and known values of κ and $\bar{P}$, it is possible to calculate $\bar{D}_1$ and $\bar{D}_2$:

$$\bar{D}_2 = \frac{c_2 \bar{P}}{\bar{t}_1 \bar{c}_2 \left(1 - z_2 / z_1\right)} \quad \text{(S6)}$$

$$\bar{D}_1 = \frac{\kappa RT/F^2 - z_2^2 \bar{D}_2 \bar{c}_2}{z_1 Q - z_1 z_2 \bar{c}_2} \quad \text{(S7)}$$

the Donnan constant, K_D, is needed to relate the coion concentrations in the solution and membrane; in its approximate form, the Donnan equation reads [4,5]: $\bar{c}_2 = \frac{K_D}{Q} c_2^2$; $\bar{c}_1$ can be found using the local electroneutrality condition ($z_1\bar{c}_1 + z_2\bar{c}_2 = Q$).

Note that $\bar{P}$ is the differential diffusion permeability, while the experiment gives the integral diffusion permeability, *P*. It is possible to convert the concentration dependence of *P(c)* into $\bar{P}(c)$ [4]:

$$\bar{P}(c) = P(c) + d\lg P / d\lg c \quad \text{(S8)}$$

and then calculate $\bar{D}_1$ and $\bar{D}_2$.

The comparison of experimental and simulated concentration dependencies of κ and P is shown in Figure 5 (see the main text of the article). Since the value of κ is very close for the AMX in KCl and NH_4Cl solutions, the Cl^- diffusion coefficient, $\bar{D}_1$, in the membrane was taken the same for both cases, $\bar{D}_{Cl^-}$ =2.7×10^{-11} m^2/s (Table 1). Since in the solution the diffusion coefficients of NH_4^+ and Cl^- are nearly identical, we assume the egality of these coefficients in the membrane: $\bar{D}_{K^+} = \bar{D}_{NH_4^+}$ =2.7×10^{-11} m^2/s (Table 1).

The concentration of fixed groups in the pore solution of the AMX membrane is calculated from the value of the exchange capacity of this membrane в Cl^- form by dividing the latter by the membrane porosity *p*=0.3; the obtained value is Q= 7.6±1.0 mol/dm^3 H_2O [6], Table 1. The value of ε is taken as 80 in solution [7] and 30 in the membrane [8].

References

1. Lévêque, A. Les Lois de La Transmission de Chaleur Par Convection. *Ann. des Mines* **1928**, *13*, 201–299.

2. Simons, R. Electric Field Effects on Proton Transfer between Ionizable Groups and Water in Ion Exchange Membranes. *Electrochim. Acta* **1984**, *29*, 151–158, doi:10.1016/0013-4686(84)87040-1.

3. Zabolotskii, V.I.; Shel'deshov, N.V.; Gnusin, N.P. Dissociation of Water Molecules in Systems with Ion-Exchange Membranes. *Russ. Chem. Rev.* **1988**, *57*, 801–808, doi:10.1070/RC1988v057n08ABEH003389.

4. Zabolotsky, V.I.; Nikonenko, V.V. Effect of Structural Membrane Inhomogeneity on Transport Properties. *J. Memb. Sci.* **1993**, *79*, 181–198, doi:10.1016/0376-7388(93)85115-D.

5. Nikonenko, V. V.; Yaroslavtsev, A.B.; Pourcelly, G. Ion Transfer in and Through Charged Membranes: Structure, Properties, and Theory. In *Ionic Interactions in Natural and Synthetic Macromolecules*; John Wiley & Sons, Inc.: Hoboken, NJ, USA, 2012; pp. 267–335 ISBN 9780470529270.

6. Sarapulova, V.; Shkorkina, I.; Mareev, S.; Pismenskaya, N.; Kononenko, N.; Larchet, C.; Dammak, L.; Nikonenko, V.; Sarapulova; Shkorkina; et al. Transport Characteristics of Fujifilm Ion-Exchange Membranes as Compared to Homogeneous Membranes AMX and CMX and to Heterogeneous Membranes MK-40 and MA-41. *Membranes (Basel).* **2019**, *9*, doi:10.3390/membranes9070084.

7. Lide, D.R. *CRC Handbook of Chemistry and Physics Editor-in-Chief*; CRC Press LLC, **2005**;

8. Kamcev, J.; Paul, D.R.; Freeman, B.D. Ion Activity Coefficients in Ion Exchange Polymers: Applicability of Manning's Counterion Condensation Theory. *Macromolecules* **2015**, *48*, 8011–8024, doi:10.1021/acs.macromol.5b01654.
